# Non-linear association between Mediterranean diet and depressive symptom in U.S. adults: A cross-sectional study

**DOI:** 10.3389/fpsyt.2022.936283

**Published:** 2022-07-15

**Authors:** Yaohua Fan, Lijun Zhao, Zhiyuan Deng, Mengzhu Li, Zifeng Huang, Meiling Zhu, Wenhua Xu

**Affiliations:** ^1^Shenzhen Hospital of Integrated Traditional Chinese and Western Medicine, Guangzhou University of Chinese Medicine, Shenzhen, China; ^2^Gaozhou Hospital of Traditional Chinese Medicine, Gaozhou, China

**Keywords:** Mediterranean diet, depressive symptom, weighted generalized additive model, random forest, multivariate logistic regression models

## Abstract

The Mediterranean diet (MED), a dietary pattern rich in fruits and vegetables, whole grains, legumes, nuts, fish, and olive oil, has anti-oxidative and anti-inflammatory effects. Although some data suggest that MED adherence is associated with decreased manifestation of depressive symptoms, it remains necessary to further analyze this apparent non-linear association as well as the influence of different factors on the relationship between MED and depression. Here, we investigated associations between the alternate MED (aMED) score and depressive symptom *via* multivariate logistic regression, weighted generalized additive (GAM) and two-step linear regression models, analyzing data from the 2005–2018 National Health and Nutrition Examination Survey (NHANES). The most important factor relevant to aMED score that contributed to the prevalence of depressive symptom was assessed using random forest. Furthermore, we examined whether the relationship between aMED score and depressive symptom differs by age, race, sex, socioeconomic variables, lifestyle- and health-related variables, and chronic medical conditions, *via* subgroup analyses. A total of 19,477 participants (20–80 years of age) were included in this cross-sectional study. In crude and adjusted (1–5) multivariate logistic regression models, increased aMED score was noted to associate with non-depressive status, as defined using the Patient Health Questionnaire-9 (*P* < 0.05). Data analyses *via* GAM and two-piecewise linear regression revealed a non-linear association between aMED and depressive symptom, which had an inflection point of 3. Random forest results revealed that vegetable score contributes greatest to the relationship between aMED and depressive symptom. Subgroup analyses revealed that aMED score is significantly negatively related with depressive symptom in most different populations (*P* < 0.05) with the exception of high annual income, diabetes, borderline blood glucose level and Parkinson's disease (PD) (*P* > 0.05). In conclusion, we observed a non-linear association between aMED score and depressive symptom. Further studies are needed to validate our results.

## Introduction

Depression, a common mental disorder, is characterized by persistent sadness and anhedonia in the context of previously rewarding or enjoyable activities (1). It is estimated that 5% of adults suffer from depression worldwide (2). The effectiveness of current prevention and treatment methods for depression, however, is limited and frequently associated with side effects. Interestingly, plant-based dietary patterns have been reported to greatly influence mental health, suggesting that diet has great use as an important adjunct therapy in the clinical management of depression (3).

Adherence to healthy diets is important to reduce the risk of illness throughout life. The Mediterranean diet (MED), which is rich in fruits and vegetables, whole grains, legumes, nuts, fish, and olive oil, is widely considered to beneficially impact health and longevity (4, 5). Not only has MED been incorporated into the national dietary guidelines set forth by the United States Department of Agriculture (USDA) but it has also attracted attention as a lifestyle modification with significant potential to reduce risks of menopausal metabolic syndrome, polycystic ovary syndrome, type 2 diabetes and cognitive decline (6–9). Furthermore, several studies reported that MED is associated with a decreased risk of depression (10–12).

However, there are several unaddressed issues in previous studies exploring MED and depression. Firstly, the alternate Mediterranean Diet (aMED) score, an important indicator for assessing adherence to MED, is a continuous variable that warrants further study to confirm its non-linear relationship with depression. Secondly, the etiology of depression is complicated and includes social, genetic and psychological factors (13, 14). Previous studies reported that the manifestation and severity of depression are not only affected by factors such as gender, educational level, marital status, and income but also depend on other comorbid conditions including stroke, thyroid problems and Parkinson's disease (PD) (15–20). Importantly, a single nucleotide variation in the oxoglutarate dehydrogenase-like gene rs2293239 (p.Asn725Ser) was identified as a major genetic predictor of familial depression (21). Thus, it is necessary to further analyze the influence of different factors on the relationship between MED and depression. Finally, prior reports warrant more detailed clarification of dietary habit influences on mental health in order to provide a foundation for effective lifestyle intervention guidelines (22).

In this study, we hypothesized aMED score and depressive symptom may have a non-linear relationship. We investigated associations between aMED score and depressive symptom *via* multivariate logistic regression, weighted generalized additive model (GAM) and two-step linear regression models, analyzing data from a large sample compiled in the National Health and Nutrition Examination Survey (NHANES) 2005–2018. Then, we identified the most important aMED score factor that contributed to depressive symptom prevalence utilizing random forest. We also examined whether the relationship between aMED score and depressive symptom differs by age, race, sex, socioeconomic variables, lifestyle- and health-related variables, and chronic medical conditions, utilizing subgroup analyses.

## Materials and methods

### Data source

The NHANES was a nationwide, cross-sectional survey conducted by the National Center for Health Statistics (NCHS). The survey included demographic, socioeconomic, dietary and health-related questions and assessed medical, dental, and physiological parameters. Laboratory tests were evaluated by appropriately trained medical personnel. Further details regarding the data collection process and analytical guidelines are available on the NHANES website (https://www.cdc.gov/nchs/nhanes/index.htm). All studies involving human participants were reviewed and approved by the Research Ethics Committee of the National Public Health Institute. Written informed consent to participate in this study was provided by the participants' legal guardian or next of kin (https://www.cdc.gov/nchs/nhanes/irba98.htm accessed on 09 December 2021).

### Study population

The 2005–2018 NHANES assessed for depressive symptom and dietary patterns among survey participants. Seven biennial datasets were combined for subsequent analysis. In this study, we excluded participants with missing dietary (*N* = 16,677) or depressive symptom (*N* = 32,730) data. In total, 19,477 participants aged over 20 years were eligible for study inclusion (Supplementary Figure 1).

### Mediterranean diet index

Dietary intake was assessed *via* structured interview focused on 24-h diet recall. The first diet recall was obtained during in-person assessment at a mobile examination center (day 1) and the second over the telephone (day 2) within 10 days of the in-person assessment. For primary analyses, we used the 24-h diet recall obtained during the in-person interview (day 1 and day 2 recalls). The MED index was determined in two steps. First, average 24-h diet recall data was linked to the USDA Food Patterns Equivalents Database to convert foods and beverages to equivalent USDA food pattern components (23). All dietary intake data from both 24-h recalls were aggregated as an average intake over 2 days for each participant. A MED index was subsequently calculated using the aMED score (4, 5). The aMED score (total score = 18) includes nine components: vegetables, legumes, fruits, nuts, whole grains, red and processed meats, fish, alcohol and olive oil (5).

### Depressive symptom

Depressive symptom was assessed using the Patient Health Questionnaire-9 (PHQ-9), a reliable and valid screening instrument composed of nine items assessing for presence and severity of clinical depressive symptoms over the past 2 weeks (24). Each of the nine items can be scored from 0 (“not at all”) to 3 (“nearly every day”), with a total score ranging from 0 to 27. Depressive symptom was dichotomized based on a PHQ-9 score ≥10. This cutoff point, frequently used in prior studies, has been noted to have a sensitivity of 88% and a specificity of 88% for detecting major depression (12, 16, 24, 25).

### Potential covariates

Baseline sociodemographic data, including demographic and questionnaire data, were obtained from NHANES. These data included sex (male or female), age (continuous; NHANES coded individuals over the age of 80 years as simply 80 years old), body mass index (BMI; <25, 25 to <30, ≥30), race (Hispanic, non-Hispanic), education (less than secondary and secondary, higher than secondary), marital status (married/living with a partner, widowed/divorced/separated, never married), annual income (< $75,000, ≥$75,000), health insurance (yes or no), self-reported health (excellent/very good, good, fair/poor), smoking history (yes or no) and insomnia (yes or no). Metabolic equivalent for task (MET) was used to assess the leisure time physical activities of participants. Self-reported time spent in moderate and vigorous leisure time exercise in a typical week was multiplied by the respective assigned MET score and values from both activities summed up to yield a total MET in mins/week. Participants who had MET-mins/week scores of ≥600 were classified as active while those with scores <600 were classified as inactive (16). Sedentary behavior was assessed by self-reported hours spent sitting at a desk, traveling in a car or bus, reading, playing cards, watching television, or using a computer on a typical day.

Medical record data, including data concerning hypertension, diabetes, stroke, thyroid illness, malignancy, cardiovascular diseases (CVD; including congestive heart failure, coronary heart disease, angina pectoris and heart attack), respiratory diseases (including emphysema, chronic bronchitis, and asthma) and PD was also collected. Many patients with PD suffered depression at the time of diagnosis. To explore whether PD was a potential covariate that affected the relationship between aMED and depressive symptom, patients suffering PD were identified amongst our participant pool (26, 27). Here, PD cases were identified if clinical history was remarkable for treatment with any of the following PD-specific medications: benztropine (generic drug code: d00175), carbidopa (generic drug code: d03473), levodopa (generic drug code: d03473), ropinirole (generic drug code: d04215), methyldopa (generic drug code: d00133), entacapone (generic drug code: d04460), and amantadine (generic drug code: d00086) (26, 27).

### Statistical analysis

The Strengthening the Reporting of Observational Studies in Epidemiology (STROBE) cross-sectional checklist was considered in this study (Supplementary Table 1) (28). Continuous variables were reported as mean ± standard deviation while categorical variables were reported as frequencies or percentages. Moreover, a complex and multi-level probability sampling design was implemented for NHANES data. As such, appropriate weighting methodology was applied in our study. Weighted Student's *t* (continuous variables included age and aMED score) and weighted chi-square (categorical variables included race, education, marital status, annual income, health insurance, self-reported health history, smoking history and insomnia, hypertension, CVD, respiratory diseases, diabetes, stroke, thyroid illness, malignancy and PD) tests were performed to assess for significant differences among means and proportions of the two groups.

Multivariate logistic regression models were weighted and generated to evaluate the association between aMED score and depressive symptom. Considering STROBE, we simultaneously obtained results of crude (no adjustment for covariates) and model 1 (only adjustment for age and sex), 2 (adjustment for socioeconomic variables), 3 (adjustment for lifestyle- and health-related variables), 4 (adjustment for chronic medical conditions) and 5 (adjustment for all covariates) adjusted analyses (28).

To evaluate the potential non-linear relationship between exposure and outcome, a GAM was generated. If a non-linear relationship was detected, a two-step linear regression model was generated to assess aMED score threshold effect and depressive symptom based on a smoothing plot. The threshold level of the aMED score at which the association between depressive symptom began to change and become significantly different was evaluated using a recurrence method. The inflection point was moved along a predefined interval with the inflection point showing maximum model likelihood investigated (29).

Random forest, a common machine learning method, was applied to analyze the contribution of various elements comprising the aMED score to depressive symptom. The learning method only allows a random sample of predictor variables was considered at each tree split. This model derived consecutive decision trees using random samples of training data to predict the residuals of previous models, thus creating a combination of trees that weighted difficulty to predict events to a greater degree (30). Based on the Gini splitting index, the optimal number of splits for each individual tree, the total number of trees, and an additional shrinkage factor—which reweights the prediction contribution from each individual tree—were determined using 10-fold cross-validation.

Subgroup analysis was conducted using a stratified multivariate logistic regression model. Participants with missing data for any other covariates were excluded. All analyses were conducted using the statistical software packages R (http://www.R-project.org, The R Foundation) and EmpowerStats (http://www.empowerstats.com, X&Y Solutions, Boston, MA, USA). *P*-values <0.05 (two-sided) were considered significantly different.

### Consent to participate

All participants in this study signed written informed consent and all research procedures were approved by the National Center for Health Statistics Research Ethics Review Board (https://www.cdc.gov/nchs/nhanes/irba98.htm). As these data are public, approval of an institutional review board was not required for this study.

## Results

### Baseline participant characteristics

In total, 19,477 participants over 20 years of age were eligible for inclusion in this study. According to PHQ-9 scoring, 16,460 participants were classified as non-depressive and 3,017 participants depressive. Baseline characteristics are list in Table 1. Depressive participant aMED score (5.81 ± 2.11) was lower compared to that of non-depressive participants (6.22 ± 2.16) (*P* < 0.001). Participants who were female, Hispanic or Other, less educated, single (widowed/divorced/separated/never married), low earners, without health insurance, with a poor self-reported health status, overweight, non-smoking, inactive, with an extended period of daily sedentary behavior, as well as a history of suffering insomnia, stroke, thyroid illness, hypertension, CVD, respiratory diseases, diabetes, PD, or malignancy, were found to be more likely to suffer depression (*P* < 0.05). However, there was no difference in the average age of participants with depressive symptom compared to those who were non-depressive.

**Table 1:** Baseline characteristics of selected participants of NHANES 2005–2018 (*n* = 19,477).

**Characteristic**	**Non-depressive status (*****n*** = **16,460)**	**Depressive status (*****n*** = **3,017)**	***P*** **-value**
Total aMED score	6.22 ± 2.16	5.81 ± 2.11	<0.001
Age (years)	49.25 ± 17.99	49.39 ± 16.32	0.692
Gender, *n* (%)			<0.001
Female	9,081 (55.17%)	1,886 (62.51%)	
Male	7,379 (44.83%)	1,131 (37.49%)	
Race, *n* (%)			<0.001
Hispanic	3,829 (23.26%)	809 (26.81%)	
Non-Hispanic	11,064 (67.22%)	1,980 (65.63%)	
Others	1,567 (9.52%)	288 (7.56%)	
Education, *n* (%)			<0.001
High school and less than high school	7,294 (44.34%)	1,775 (58.85%)	
More than high school	9,156 (55.66%)	1,241 (41.15%)	
Marital status, *n* (%)			<0.001
Married/living with partner	9,831 (59.76%)	1,415 (46.93%)	
Widowed/divorced/ separated	3,581 (21.77%)	997 (33.07%)	
Never married	3,040 (18.48%)	603 (20.00%)	
Annual income, *n* (%)			<0.001
<75,000$	12,149 (76.97%)	2,557 (89.59%)	
≥75,000$	3,635 (23.03%)	297 (10.41%)	
Health insurance, *n* (%)			<0.001
Yes	13,163 (80.04%)	2,272 (75.38%)	
No	3,282 (19.96%)	742 (24.62%)	
Self-reported health status, ***n*** (%)			<0.001
Excellent/very good	5,623 (34.16%)	333 (11.04%)	
Good	6,932 (42.11%)	953 (31.59%)	
Fair/poor	3,905 (23.72%)	1,731 (57.37%)	
BMI category (km/m^2^)			<0.001
<25.0	4,510 (27.63%)	703 (23.62%)	
25.0 to <30.0	5,262 (32.24%)	770 (25.87%)	
≥30.0	6,549 (40.13%)	1,503 (50.50%)	
Smoking, *n* (%)			<0.001
Yes	9,084 (55.22%)	1,245 (41.27%)	
No	7,367 (44.78%)	1,772 (58.73%)	
Leisure-time physical activity, ***n*** (%)			<0.001
Active	10,567 (67.51%)	1,612 (56.98%)	
Quiet	5,085 (32.49%)	1,217 (43.02%)	
Sedentary time (hours/day), ***n*** (%)			0.009
≥ 6	7,397 (44.94%)	1,433 (47.50%)	
<6	9,063 (55.06%)	1,584 (52.50%)	
Trouble sleeping, *n* (%)			<0.001
Yes	4,836 (29.39%)	1,728 (57.28%)	
No	11,620 (70.61%)	1,289 (42.72%)	
Stroke, *n* (%)			<0.001
Yes	625 (3.80%)	238 (7.92%)	
No	15,813 (96.20%)	2,768 (92.08%)	
Thyroid problem, *n* (%)			<0.001
Yes	1,876 (11.41%)	484 (16.16%)	
No	14,559 (88.59%)	2,511 (83.84%)	
Hypertension, *n* (%)			<0.001
Yes	6,024 (36.63%)	1,446 (47.99%)	
No	10,420 (63.37%)	1,567 (52.01%)	
CVD, *n* (%)			<0.001
Yes	1,412 (8.58%)	454 (15.06%)	
No	15,045 (91.42%)	2,561 (84.94%)	
Respiratory diseases, *n* (%)			<0.001
Yes	3,233 (19.64%)	958 (31.75%)	
No	13,226 (80.36%)	2,059 (68.25%)	
Diabetes, *n* (%)			<0.001
Yes	2,162 (13.14%)	596 (19.79%)	
No	13,899 (84.50%)	2,324 (77.18%)	
Borderline	388 (2.36%)	91 (3.02%)	
Parkinson's disease, *n* (%)			
Yes	139 (0.84%)	67 (2.22%)	<0.001
No	16,321 (99.16%)	2,950 (97.78%)	
Cancer or malignancy, *n* (%)			0.006
Yes	1,636 (9.95%)	349 (11.58%)	
No	14,807 (90.05%)	2,664 (88.42%)	

*aMED, Alternate Mediterranean Diet score; BMI, body mass index*.

### Multiple logistic regression analyses of aMED score and depressive symptom

Multivariable logistic regression was applied to evaluate the relationship between aMED score and the prevalence of depressive symptom (Table 2). In the crude model, the prevalence of depressive symptom and aMED score were negatively associated (OR: 0.915, 95% CI: 0.899–0.932; *P* < 0.001). In the adjusted model 1 (adjusted for age, sex and race), results did not differ from those obtained using crude analysis (OR: 0.905, 95% CI: 0.888–0.922; *P* < 0.001). After adjusting for different covariate categories in models 2–5, a negative association between depressive symptom and aMED score remained (Table 2). Our findings indicated that higher aMED score was associated with lower odds of depressive symptom; results were stable and robust.

**Table 2:** Multivariable logistic regression of the association between aMED score and the prevalence of depressive symptom.

**Outcome**	**Crude model**	**Model 1**	**Model 2**	**Model 3**	**Model 4**	**Model 5**
	**OR (95% CI)** ***P*****-value**	**OR (95% CI)** ***P*****-value**	**OR (95% CI)** ***P*****-value**	**OR (95% CI)** ***P*****-value**	**OR (95% CI)** ***P*****-value**	**OR (95% CI)** ***P*****-value**
Prevalence of depressive symptom	0.915 (0.899, 0.932) <0.001	0.905 (0.888, 0.922) <0.001	0.935 (0.917, 0.953) <0.001	0.929 (0.905, 0.955) <0.001	0.922 (0.905, 0.939) <0.001	0.971 (0.950, 0.993) 0.009

*Crude model adjusted for: none*.

*Model 1 adjusted for: age, gender and race*.

*Model 2 adjusted for: socioeconomic variables including education, marital status, annual income, and health insurance*.

*Model 3 adjusted for: lifestyle- and health-related variables including BMI category, self-reported health status, leisure-time physical activity, sedentary time, and trouble sleeping*.

*Model 4 adjusted for: chronic medical conditions including stroke, thyroid problem, hypertension, diabetes, CVD, respiratory diseases, Parkinson's disease, cancer or malignancy*.

*Model 5 adjusted for: all covariates listed in Table 1 were adjusted*.

*OR, odds ratio; CI, confidence interval*.

### Analysis of non-linear relationships between aMED scores and depressive symptom

**Figure 1:**
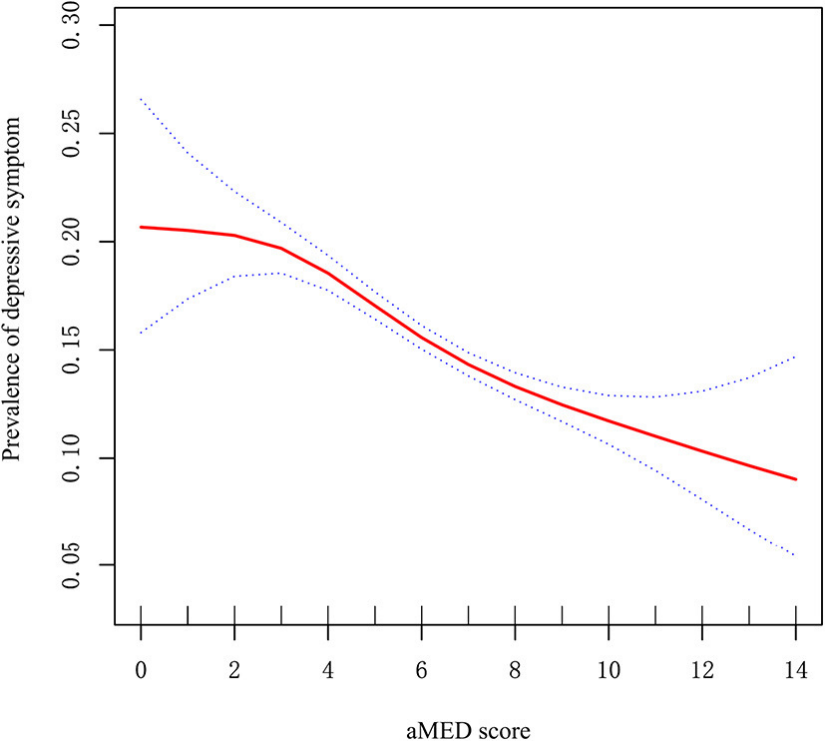
Non-linear relationship between aMED score and prevalence of depressive symptom. X-axis: aMED score; Y-axis: prevalence of depressive symptom. Red: OR; blue circles: 95% CI.

Because aMED score is a continuous variable, we considered that it may have a non-linear relationship with depressive symptom. *Via* GAM, we found a non-linear relationship between aMED score and depressive symptom (Figure 1). Using two-step linear regression analysis, we calculated an inflection point of 3. To the left of the inflection point, effect size, 95% CI and *P*-values were 1.079, 0.924–1.259, and 0.337, respectively. A negative relationship, however, was also observed between aMED score and depressive symptom to the right of the inflection point (0.907, 0.889–0.926, and <0.001; (Supplementary Table 2).

### Random forest analysis of aMED score component influence on depressive symptom

To explore which aMED score components contributed most to the prevalence of depressive symptom, we used a random forest method for further analysis. High to low component contributions were as follows: vegetable, fruit, fish, meat, alcohol, legume, dairy, and cereal scores (Supplementary Figure 2). These results underscored a stronger association between high-fiber foods and a lower prevalence of depressive symptom.

### Results of subgroup analyses

Because many factors are associated with depression, we performed a stratified analysis of populations with different characteristics to identify factors that influence the relationship between aMED score and depressive symptom. As shown in Supplementary Table 3, the negative relationship between aMED score and depressive symptom does not change in most characteristic populations irrespective of factors such as age, sex, race, education level, marital status, health insurance, BMI, smoking, leisure time physical activity, sedentary behavior, insomnia, stroke, thyroid illness, hypertension, CVD, respiratory diseases or malignancy (*P* < 0.001). Interestingly, no significant associations between aMED score and depressive symptom were observed among high annual income earners (*P* = 0.093), diabetics (*P* = 0.063), individuals with borderline blood glucose levels (*P* = 0.093), and patients suffering PD (*P* = 0.474).

## Discussion

Over the past several decades, interest concerning the prevention and treatment of diseases *via* dietary modification has shifted from studying the effects of single nutrients and foods to studying dietary patterns (31). A meta-analysis of randomized controlled trials suggested that dietary interventions hold promise for effectively reducing symptoms of depression (22). Adherence to MED in particular appears to reduce manifestation of chronic illnesses and premature mortality (32). Although evidence of benefits concerning a lower prevalence of depression in the setting of proper MED adherence is scarce, some studies have reported such findings (10–12). Our results similarly suggest a negative association between aMED score and the prevalence of depressive symptom. On the basis of multivariate logistic regression analysis, we applied GAM to further evaluate the non-linear association between aMED score and the prevalence of depressive symptom. Importantly, GAM can not only address non-parametric smoothing but also fit a regression spline to data, which is obviously advantageous in analysis of non-linear associations (33). Here, we noted a negative association between aMED score and the prevalence of depressive symptom at an aMED score of 3 points or greater. These findings suggest that individuals with depressive symptoms should ideally strive for aMED scores of >3, thus increasing the likelihood that MED alleviates clinical depression.

The beneficial effects of the MED can be mainly attributed to its numerous components rich in anti-oxidants and possessing anti-inflammatory properties (34). Positive effects in the setting of depression are likely mainly due to the activity of unsaturated fatty acids and polyphenols (2018). Moreover, our findings suggest that high-fiber food, including vegetables and fruit, plays a greater role than foods such as fish, meat, alcohol, legumes, dairy and cereals, with respect to the negative association between aMED score and depressive symptom. These results demonstrate MED is a dietary pattern based on plant-derived foods (32). Importantly, vegetables and fruits are rich in phytochemicals, phenolic compounds and flavonoids. Most of these compounds possess significant anti-oxidant properties which likely improve depressive symptoms *via* inhibition of oxidative stress and alleviation of chronic inflammation. For example, lutein, a dietary phytochemical capable of crossing the blood-brain barrier, has been demonstrated to exert an antidepressant-like effect and alleviate neurochemical imbalances (35). Curcumin, a polyphenol extracted from the rhizome of the turmeric plant, has been demonstrated to exert antidepressant effects in both animal and human trials (36, 37). Similarly, S-equol, a metabolite of dietary soy isoflavones, was reported to alleviate depressive-like behavior in mice by inhibiting neuroinflammation and enhancing synaptic plasticity (38). Moreover, omega-3 polyunsaturated fatty acids may reduce inflammation and benefit mood by fostering the production of the anti-inflammatory prostaglandin E3 (as opposed to the pro-inflammatory prostaglandin E2) (39). Although vegetable and fruit scores were noted to exert a greater effect on the association between aMED score and the prevalence of depressive symptom, other ingredients also contribute to the relationship. As such, the anti-inflammatory and mood-related benefits of MED adherence result due to a synergistic action of nutrients composing this dietary pattern.

Due to the complex etiology of depression, there is currently no universal and effective treatment. In order to make the case for clinical application of MED in effective prevention and management of depression, we used subgroup analyses to explore the association between aMED score and the prevalence of depressive symptom in different populations. Results revealed that aMED score has a significantly negative relationship with depressive symptom in most populations, except among patients earning a high annual income or suffering diabetes, borderline blood glucose levels or PD. Previous study reported that high annual income earners generally have higher aMED scores (5). In addition, most American adults do not adhere to dietary patterns resembling MED. This phenomenon may explain the lack of a statistically significant association between aMED score and depressive symptom among high annual income earners. Furthermore, cognitive impairment is one key risk factor of depression (40). Cognitive impairment is also known to be a main complication of diabetes and PD (41, 42). This may similarly explain the lack of a statistically significant association between aMED score and depressive symptom in patients suffering diabetes or PD. Interestingly, the protective effect of MED on depressive symptoms was noted to gradually decrease as BMI increased. The prevalence of depression is known to be increased in the setting of obesity, a condition that promotes inflammation as well as resistance to insulin and leptin (43, 44). Thus, a combination of MED adherence and proper weight control is guaranteed to improve the effectiveness of MED adherence in regard to alleviating depression. No or little association of MED score with depression among younger women, however, has also been reported due to genetic factors (45). We found that age and sex do not influence the relationship between aMED score and depressive symptom. Therefore, further research is needed to confirm the aforementioned discrepancies.

There were some limitations in our study. First, due to the nature of cross-sectional design of present study, residual confounding from unmeasured confounders should be a concern. Second, dietary intake was assessed by asking subjects to recall what was eaten over the prior 2 days and thus may not accurately reflect the typical dietary patterns of subjects. Third, due to missing data, some psychiatric comorbidities were not considered as covariates in our analyses. For example, eating disorders and attention deficit hyperactivity disorder were not considered in this study (46, 47). Moreover, the state of mental health cannot be ruled out in regard to exerting a significant influence on the association between aMED score and depression as cognitive function and prescription medications were not assessed in this study. In addition, possible misclassification of dietary behavior due to memory bias may have occurred during data collection. Finally, although the PHQ-9 is a validated screening tool designed to assess depressive symptoms, it is not intended for use in the diagnosis of clinical depression. Furthermore, as diagnostic delay of depression in clinical practice is common, our findings might be caused by reversion causation; we were unable to consider this phenomenon when performing analyses due to a lack of relevant data (48). Future studies are warranted to clarify the impact of diagnostic delay on the relationship between MED and depression.

## Conclusions

In this study, the relationship between aMED score and depressive symptom was demonstrated to be non-linear. Our findings confirm that MED alleviates depressive symptom at aMED scores of 3 or greater. However, the association between aMED score and depressive symptom in populations earning a high annual income, as well as suffering diabetes, borderline blood glucose levels or PD, requires further evaluation.

## Data availability statement

The data presented in the study are deposited in the the NHANES website, accession link: https://www.cdc.gov/nchs/nhanes/index.htm.

## Ethics statement

The studies involving human participants were reviewed and approved by the Research Ethics Committee of the National Public Health Institute. Written informed consent to participate in this study was provided by the participants' legal guardian/next of kin (https://www.cdc.gov/nchs/nhanes/irba98.htm accessed on December 9, 2021).

## Author contributions

Conceptualization: WX and MZ. Methodology, validation, and writing—original draft preparation: YF. Software and visualization: ZD. Formal analysis, data curation, and writing—review and editing: LZ. Investigation: ML. Resources: ZH. Supervision and funding acquisition: MZ. Project administration: WX. All authors have read and agreed to the published version of the manuscript.

## Funding

The present study was supported by the Traditional Chinese Medicine Bureau of Guangdong Province (20211345), the Natural Science Foundation of Guangdong Province (2022A151510450), the Shenzhen Science and Technology Innovation Committee Subject (JCYJ20210324123614040), Bao'an TCM Development Foundation (2020KJCX-KTYJ-130 and 2020KJCX-KTYJ-133), and Sanming Project of Medicine in Shenzhen (SZZYSM202106009).

## Conflict of interest

The authors declare that the research was conducted in the absence of any commercial or financial relationships that could be construed as a potential conflict of interest.

## Publisher's note

All claims expressed in this article are solely those of the authors and do not necessarily represent those of their affiliated organizations, or those of the publisher, the editors and the reviewers. Any product that may be evaluated in this article, or claim that may be made by its manufacturer, is not guaranteed or endorsed by the publisher.
