# Supplementary material for: Non-linear association between Mediterranean diet and depressive symptom in U.S. adults: A cross-sectional study
Source: Front Psychiatry. 2022 Jul 15;13:936283. doi: 10.3389/fpsyt.2022.936283 (PMC9334730; doi:10.3389/fpsyt.2022.936283)
Supplement: Supplementary file 1 [file Data_Sheet_1.docx]

Supplementary Material

**Non-linear association between Mediterranean diet and depressive symptom in U.S. adults: a cross-sectional study**

**Yaohua Fan^1^, Lijun Zhao^1^, Zhiyuan Deng^2^, Mengzhu Li^1^, Zifeng Huang^1^, Meiling Zhu^1*^, Wenhua Xu^1*^**

*To whom correspondence should be addressed.

***Correspondence:**

Wenhua Xu
[xwhua2002@163.com](mailto:xwhua2002@163.com)

Meiling zhu

meilingzhu2020@126.com

# Supplementary Figures and Tables

## Supplementary Figures


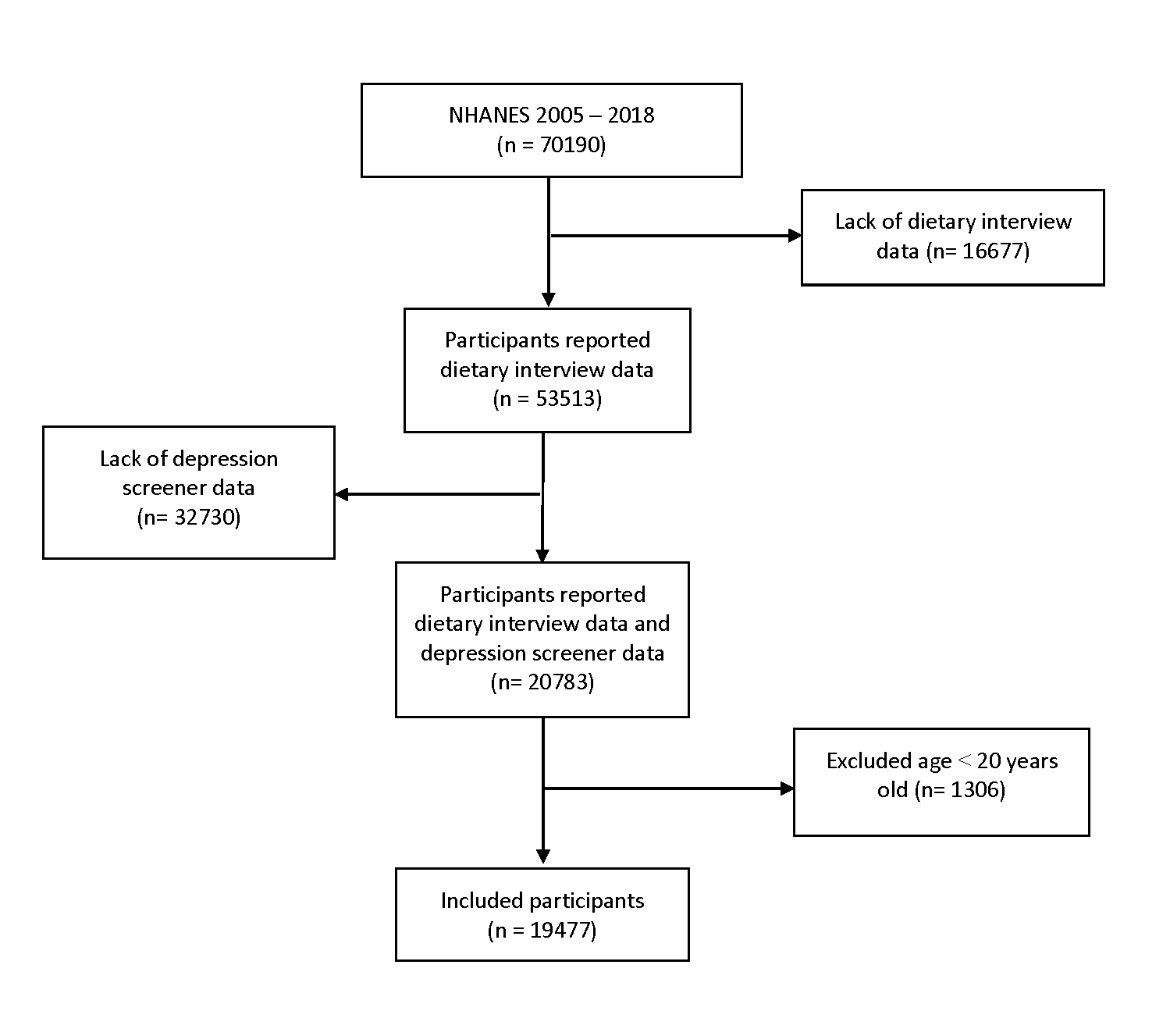


**Supplementary Figure 1.** Flowcharts illustrating sample selection from NHANES 2005–2018. NHANES; the National Health and Nutrition Examination Survey.


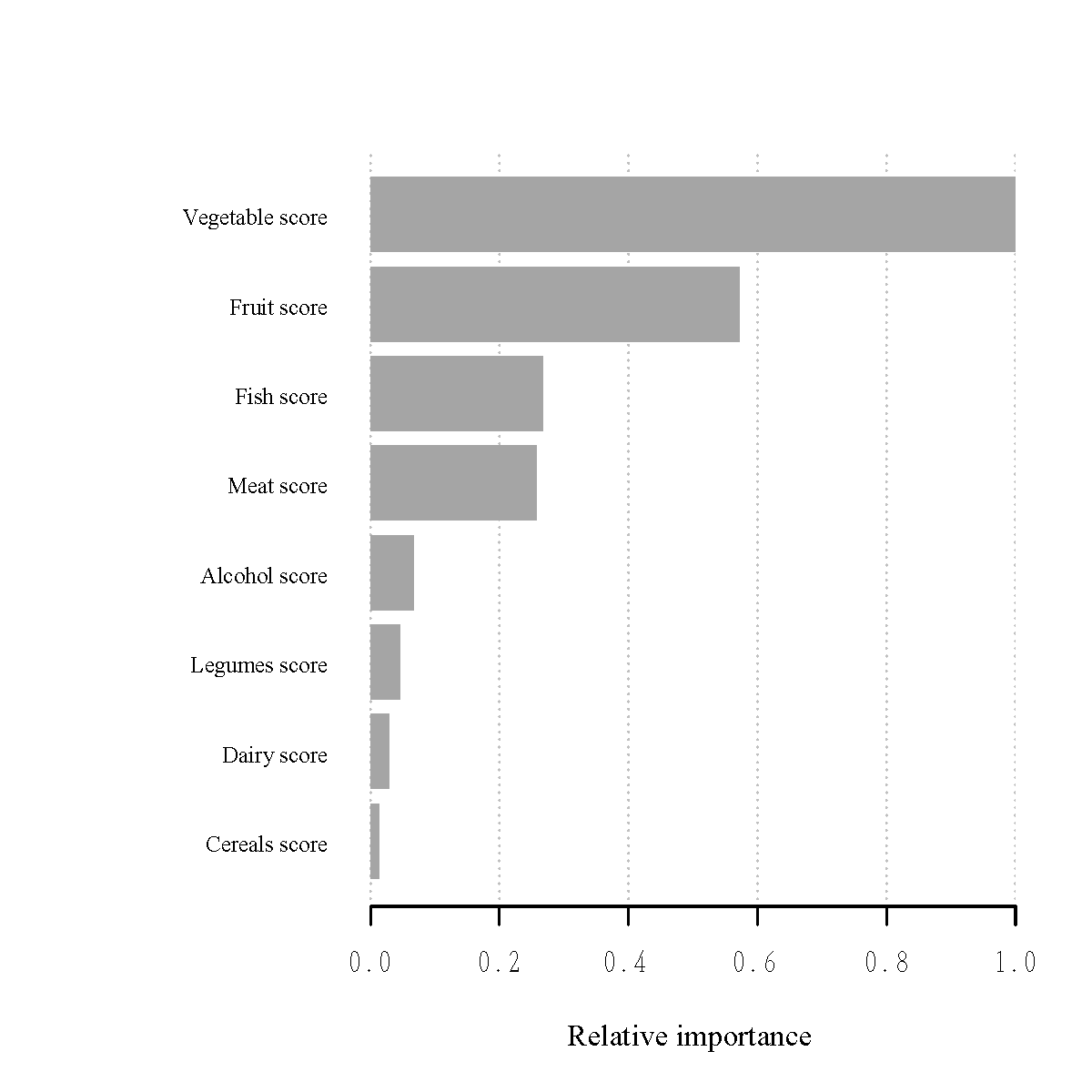


**Supplementary Figure 2.** Importance of predictor variables in random forest analysis, scaled to a maximum of 100.

## Tables

Supplementary Table 1. STROBE Statement—checklist

|  | Item No | Recommendation | Included |
| --- | --- | --- | --- |
| **Title and abstract** | 1 | (*a*) Indicate the study’s design with a commonly used term in the title or the abstract | Title |
|  |  | (*b*) Provide in the abstract an informative and balanced summary of what was done and what was found | Abstract |
| Introduction | | |  |
| Background/rationale | 2 | Explain the scientific background and rationale for the investigation being reported | Paragraph 1-3 in Introduction |
| Objectives | 3 | State specific objectives, including any prespecified hypotheses | Paragraph 4 in Introduction |
| Methods | | |  |
| Study design | 4 | Present key elements of study design early in the paper | Paragraph 1 in Materials and Methods |
| Setting | 5 | Describe the setting, locations, and relevant dates, including periods of recruitment, exposure, follow-up, and data collection | Paragraph 1 in Materials and Methods |
| Participants | 6 | (*a*) *Cross-sectional study*—Give the eligibility criteria, and the sources and methods of selection of participants | Paragraph 2 in Materials and Methods |
|  |  | (*b*) *Cohort study*—For matched studies, give matching criteria and number of exposed and unexposed  *Case-control study*—For matched studies, give matching criteria and the number of controls per case | No applicable |
| Variables | 7 | Clearly define all outcomes, exposures, predictors, potential confounders, and effect modifiers. Give diagnostic criteria, if applicable | Paragraph 3-4 in Materials and Methods |
| Data sources/ measurement | 8* | For each variable of interest, give sources of data and details of methods of assessment (measurement). Describe comparability of assessment methods if there is more than one group | Paragraph 5-6 in Materials and Methods |
| Bias | 9 | Describe any efforts to address potential sources of bias | Paragraph 6 in Materials and Methods |
| Study size | 10 | Explain how the study size was arrived at | Paragraph 11 in Materials and Methods |
| Quantitative variables | 11 | Explain how quantitative variables were handled in the analyses. If applicable, describe which groupings were chosen and why | Paragraph 5-6 in Materials and Methods |
| Statistical methods | 12 | (*a*) Describe all statistical methods, including those used to control for confounding | Paragraph 7-10 in Materials and Methods |
|  |  | (*b*) Describe any methods used to examine subgroups and interactions | Paragraph 10 in Materials and Methods |
|  |  | (*c*) Explain how missing data were addressed | Paragraph 11 in Materials and Methods |
|  |  | (*d*) *Cross-sectional study*—If applicable, describe analytical methods taking account of sampling strategy | No applicable |
|  |  | (*e*) Describe any sensitivity analyses | Paragraph 11 in Materials and Methods |

| Results | | |  |
| --- | --- | --- | --- |
| Participants | 13* | (a) Report numbers of individuals at each stage of study—eg numbers potentially eligible, examined for eligibility, confirmed eligible, included in the study, completing follow-up, and analysed | Paragraph 1 in Results |
|  |  | (b) Give reasons for non-participation at each stage | No applicable |
|  |  | (c) Consider use of a flow diagram | Supplementary figure 1 |
| Descriptive data | 14* | (a) Give characteristics of study participants (eg demographic, clinical, social) and information on exposures and potential confounders | Paragraph 1 in Results |
|  |  | (b) Indicate number of participants with missing data for each variable of interest | Paragraph 1 in Results |
|  |  | (c) *Cohort study*—Summarise follow-up time (eg, average and total amount) | No applicable |
| Outcome data | 15* | *Cohort study*—Report numbers of outcome events or summary measures over time | No applicable |
|  |  | *Case-control study—*Report numbers in each exposure category, or summary measures of exposure | No applicable |
|  |  | *Cross-sectional study—*Report numbers of outcome events or summary measures | Paragraph 1 in Results |
| Main results | 16 | (*a*) Give unadjusted estimates and, if applicable, confounder-adjusted estimates and their precision (eg, 95% confidence interval). Make clear which confounders were adjusted for and why they were included | Paragraph 2-3 in Results |
|  |  | (*b*) Report category boundaries when continuous variables were categorized | Paragraph 3-5 in Materials and Methods |
|  |  | (*c*) If relevant, consider translating estimates of relative risk into absolute risk for a meaningful time period | No applicable |
| Other analyses | 17 | Report other analyses done—eg analyses of subgroups and interactions, and sensitivity analyses | Paragraph 5 in Results |
| Discussion | | |  |
| Key results | 18 | Summarise key results with reference to study objectives | Paragraph 1 in Discussion |
| Limitations | 19 | Discuss limitations of the study, taking into account sources of potential bias or imprecision. Discuss both direction and magnitude of any potential bias | Paragraph 4 in Discussion |
| Interpretation | 20 | Give a cautious overall interpretation of results considering objectives, limitations, multiplicity of analyses, results from similar studies, and other relevant evidence | Paragraph 2-3 in Discussion |
| Generalisability | 21 | Discuss the generalisability (external validity) of the study results | Paragraph 1 in Conclusions |
| Other information | | |  |
| Funding | 22 | Give the source of funding and the role of the funders for the present study and, if applicable, for the original study on which the present article is based | Paragraph 1 in Funding |

*Give information separately for cases and controls in case-control studies and, if applicable, for exposed and unexposed groups in cohort and cross-sectional studies.

Supplementary Table 2**.** Non-linearity addressing by weighted two-piecewise linear model

|  | aMED score | |
| --- | --- | --- |
|  | OR (95%CI) | *P* value |
| Fitting by standard linear model | 0.915 (0.899, 0.932) | ＜0.001 |
| Fitting by two-piecewise linear model |  |  |
| aMED score＜3 | 1.079 (0.924, 1.259) | 0.337 |
| aMED score ≥ 3 | 0.907 (0.889, 0.926) | <0.001 |
| Log likelihood ratio | 0.032 |  |

aMED score: Alternate Mediterranean Diet score; OR: odds ratio; CI: confidence interval.

Supplementary Table 3. Effect size of aMED score on depressive symptom in exploratory subgroups

| Characteristic | Sample size | OR (95% CI) *P*-value |
| --- | --- | --- |
| Age (years) |  |  |
| <40 | 6609 | 0.911 (0.882, 0.942) <0.001 |
| 40 to <60 | 6490 | 0.902 (0.875, 0.929) <0.001 |
| ≥60 | 6378 | 0.938 (0.908, 0.970) <0.001 |
| Gender |  |  |
| Female | 10967 | 0.914 (0.892, 0.936) <0.001 |
| Male | 8510 | 0.905 (0.879, 0.932) <0.001 |
| Race |  |  |
| Hispanic | 4638 | 0.952 (0.919, 0.986) 0.006 |
| Non-Hispanic | 13044 | 0.894 (0.874, 0.915) <0.001 |
| Others | 1795 | 0.908 (0.851, 0.967) 0.003 |
| Education |  |  |
| High school and less than high school | 9069 | 0.952 (0.929, 0.976) <0.001 |
| More than high school | 10397 | 0.896 (0.871, 0.922) <0.001 |
| Marital status |  |  |
| Married/Living with partner | 11246 | 0.904 (0.880, 0.928) <0.001 |
| Widowed/Divorced/Separated | 4578 | 0.941 (0.910, 0.973) <0.001 |
| Never married | 3643 | 0.924 (0.886, 0.963) <0.001 |
| Annual income |  |  |
| ＜75000＄ | 14706 | 0.920 (0.902, 0.939) <0.001 |
| ≥ 75000＄ | 3932 | 0.954 (0.903, 1.008) 0.093 |
| Health insurance |  |  |
| Yes | 15435 | 0.913 (0.894, 0.932) <0.001 |
| No | 4024 | 0.933 (0.900, 0.968) <0.001 |
| Self-reported health |  |  |
| Excellent/very good | 5956 | 0.911 (0.865, 0.960) <0.001 |
| Good | 7885 | 0.953 (0.923, 0.984) 0.003 |
| Fair/poor | 5636 | 0.948 (0.923, 0.974) <0.001 |
| BMI category (km/m2) |  |  |
| <25.0 | 5213 | 0.891 (0.859, 0.924) <0.001 |
| 25.0 to <30.0 | 6032 | 0.907 (0.875, 0.940) <0.001 |
| ≥30.0 | 8052 | 0.948 (0.923, 0.974) <0.001 |
| Smoking |  |  |
| Yes | 9139 | 0.918 (0.896, 0.941) <0.001 |
| No | 10329 | 0.943 (0.917, 0.970) <0.001 |
| Leisure-time physical activity |  |  |
| Active | 12179 | 0.917 (0.895, 0.940) <0.001 |
| Inactive | 6302 | 0.916 (0.888, 0.944) <0.001 |
| Sedentary time (hours/day) |  |  |
| ≥6 | 8830 | 0.916 (0.891, 0.941) <0.001 |
| <6 | 10647 | 0.915 (0.893, 0.938) <0.001 |
| Trouble sleeping |  |  |
| Yes | 6564 | 0.923 (0.899, 0.948) <0.001 |
| No | 12909 | 0.920 (0.896, 0.945) <0.001 |
| Stroke |  |  |
| Yes | 863 | 0.901 (0.837, 0.969) 0.005 |
| No | 18581 | 0.918 (0.901, 0.936) <0.001 |
| Thyroid problem |  |  |
| Yes | 2360 | 0.875 (0.834, 0.918) <0.001 |
| No | 17070 | 0.920 (0.902, 0.938) <0.001 |
| Hypertension |  |  |
| Yes | 7470 | 0.924 (0.899, 0.950) <0.001 |
| No | 11987 | 0.912 (0.890, 0.935) <0.001 |
| CVD |  |  |
| Yes | 1866 | 0.909 (0.865, 0.955) <0.001 |
| No | 17606 | 0.918 (0.900, 0.936) <0.001 |
| Respiratory diseases |  |  |
| Yes | 4191 | 0.898 (0.867, 0.930) <0.001 |
| No | 15285 | 0.929 (0.909, 0.950) <0.001 |
| Diabetes |  |  |
| Yes | 2758 | 0.961 (0.921, 1.002) 0.063 |
| No | 16223 | 0.908 (0.889, 0.927) <0.001 |
| Borderline | 479 | 0.908 (0.811, 1.016) 0.093 |
| Parkinson's disease |  |  |
| Yes | 206 | 1.052 (0.916, 1.209) 0.474 |
| No | 19271 | 0.913 (0.896, 0.930) <0.001 |
| Cancer or malignancy |  |  |
| Yes | 1985 | 0.902 (0.854, 0.954) <0.001 |
| No | 17471 | 0.917 (0.899, 0.935) <0.001 |

Supplementary Table S3. The confusion matrix of the cross-validation.txt. (Supplementary Table S3.xls)
